# Annual Commencement of the Buffalo Medical College

**Published:** 1877-03

**Authors:** 


### Annual Commencement of the Buffalo Medical College.

The annual examination before the Faculty and Curators, was held Tuesday, February 20th, 1877, resulting in the graduation as *Doctors in Medicine* of the following gentlemen:

THE GRADUATING CLASS.

Arthur Martin Barker, Buffalo, N. Y.; James Henry Tamblin, Black River, N. Y.; Joseph Haberstro, Buffalo, N. Y.; George Heman Saddleson, Cambria, N. Y.; Edward Eckerson, Shelby, N. Y.; Charles Shupe, Font Hill, Ontario; John Warner Huntington, Mexico, N. Y.; Francis Waters Gallagher, Buffalo, N. Y.; Sylvenus Emmons Parker, Sanborn, N. Y.; Seldon Johnson Mudge, Olean, N. Y.; William Francis Sheehan, Rochester, N. Y.; Edward N. B. Mershon, Youngstown, Pa.; Lee Herbert Smith, Buffalo, N. Y.; Daniel Williams Allen, Buffalo, N. Y.; Cyrus C. Harvey, Dundee, N. Y.; John Emery Copp, Sinclairville, N. Y.; Fred. Liona Gould, Frewsburg, N. Y.; Arthur Edson Blair, Castle Creek, N. Y.; John Howard Miller, Rouseville, Pa.; Frank Eugene Cruttenden, Bath, N. Y.; Henry Amos Wilmot, Spencerport, N. Y.; Charles Edgar Heaton, Mexico, N. Y.; Frank Guy Sherwood, Medina, N. Y.; John R. Mullen, Alexander, N. Y.; George S. Wyckoff, Buffalo, N.; Louis Charles Volker, Buffalo, N. Y.; Francis B. Kendrick, Buffalo, N. Y.; Arthur Lee Lowe, Corry, Pa.; John J. A. Burke, Rochester, N. Y.; Edwin Richard Hopkins, Westfield, N. Y.

The examination was highly satisfactory, the whole class acquitting itself very creditably.

The public exercises of the occasion were held in the evening, and were largely attended. After the conferring of Degrees by Prof. WHITE, Vice Chancellor of the University, Prof. E. V. STODARD delivered the Address before the Graduating Class. This was a highly interesting and instructive feature of the day. As the daily press published the various addresses of the day and evening, it is unnecessary to speak of their merits or republish them. If we had expected to select the material for this Journal at that time, we should have provided for it.

After the conferring of Degrees, Prof. ROCHESTER, Dean of the Faculty, announced that prizes had been given in the College during the past few years for excellence in certain branches of study, and he took pleasure in making public the names of the successful competitors and presenting the prizes, which he did as follows:

*The Fillmore Prize.*—1st, $30. Awarded to Wm. F. Sheehan, of Rochester, N. Y.

2d, $10. Divided between Arthur M. Barker and Lee H. Smith of Buffalo.

Honorable mention was made of Francis W. Gallagher, of Buffalo.

Committee—Profs. Stoddard and Moore.

*The Prof. White Prize.*—A complete Obstetrical case of instruments. Awarded to Francis W. Gallagher, of Buffalo. Motto: "Ministry of Buffalo."

Committee—E. N. Brush, M. D., E. C. W. O'Brien, M. D., W. W. Miner, M. D.

*The Prof. Rochester Prize.*—A complete Stethoscopic case. Awarded to Wm. F. Sheehan, of Rochester, N. Y. Motto: "*Pro Bono Publico.*"

Honorable mention was made of Thomas M. Rochester, of Rochester. Motto: "Marius."

Committee—Frank W. Abbott, M. D., W. C. Phelps, M. D.

*The Prof. Moore Prize.*—A copy of "Holmes' Surgery." Awarded to Wm. F. Sheehan, of Rochester.

*The Prof. Miner Prize.*—A complete miner pocket case. Awarded to Thomas M. Rochester, of Rochester, an under graduate. Motto: "Marius."

*The Prof. Potter Prize.*—A complete anatomical case. Awarded to Francis W. Gallagher, of Buffalo.

*The Prof Stoddard Prize.*—Stille's "Therapeutics and *Materia Medica*"—2 vols. Awarded to Lee H. Smith, of Buffalo.

Honorable mention was made of Wm. F. Sheehan, of Rochester, and of Arthur M. Baker of Buffalo.

It is almost needless to state that the friends of the young gentlemen cheered them warmly as they were called out. After some music came the address to the graduates, by Prof. E. V. STODDARD, which on account of its rare merit we will publish in our next Journal, if we can secure a copy, the public not having obtained it.

